# A model for self-organization of sensorimotor function: the spinal monosynaptic loop

**DOI:** 10.1152/jn.00242.2021

**Published:** 2022-03-09

**Authors:** Jonas M. D. Enander, Adam M. Jones, Matthieu Kirkland, Jordan Hurless, Henrik Jörntell, Gerald E. Loeb

**Affiliations:** ^1^Department of Experimental Medical Science, Faculty of Medicine, Lund University, Lund, Sweden; ^2^Department of Biomedical Engineering, Viterbi School of Engineering, University of Southern California, Los Angeles, California

**Keywords:** extrafusal muscle, intrafusal muscle, muscle spindle, neuron model, spinal development

## Abstract

Recent spinal cord literature abounds with descriptions of genetic preprogramming and the molecular control of circuit formation. In this paper, we explore to what extent circuit formation based on learning rather than preprogramming could explain the selective formation of the monosynaptic projections between muscle spindle primary afferents and homonymous motoneurons. We adjusted the initially randomized gains in the neural network according to a Hebbian plasticity rule while exercising the model system with spontaneous muscle activity patterns similar to those observed during early fetal development. Normal connectivity patterns developed only when we modeled β motoneurons, which are known to innervate both intrafusal and extrafusal muscle fibers in vertebrate muscles but were not considered in previous literature regarding selective formation of these synapses in animals with paralyzed muscles. It was also helpful to correctly model the greatly reduced contractility of extrafusal muscle fibers during early development. Stronger and more coordinated muscle activity patterns such as observed later during neonatal locomotion impaired projection selectivity. These findings imply a generic functionality of a musculoskeletal system to imprint important aspects of its mechanical dynamics onto a neural network, without specific preprogramming other than setting a critical period for the formation and maturation of this general pattern of connectivity. Such functionality would facilitate the successful evolution of new species with altered musculoskeletal anatomy, and it may help to explain patterns of connectivity and associated reflexes that appear during abnormal development.

**NEW & NOTEWORTHY** A novel model of self-organization of early spinal circuitry based on a biologically realistic plant, sensors, and neuronal plasticity in conjunction with empirical observations of fetal development. Without explicit need for guiding genetic rules, connection matrices emerge that support functional self-organization of the mature pattern of Ia to motoneuron connectivity in the spinal circuitry.

## Graphical abstract

**Figure:**
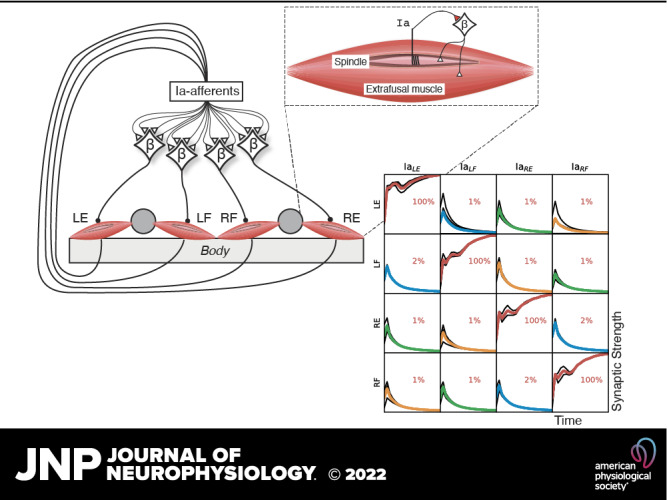

## INTRODUCTION

Motor commands from the brain are processed through the neuronal circuitry of the spinal cord, where they are integrated with sensory signals before producing output in Sherrington’s final common pathway of the motoneuron (MN) (1). Because the sensory signals depend on the musculoskeletal mechanics (the “plant” in engineering terms), the combination of spinal circuitry plus plant mechanics defines the control problems that the brain must solve to generate desired motor behaviors (2–4). It would seem to be advantageous for the spinal circuitry to reflect the plant mechanics (5), but it is not clear what that means given the complexity of both the mechanics and the circuitry in vertebrates.

The recent spinal cord literature abounds with descriptions of genetic preprogramming and the molecular control of circuit formation (6–9). The question we are asking is whether the genetic programs specify only general rules for types of connectivity, rather than being responsible for creating fully functional connectivity patterns that reflect the functional properties of the musculoskeletal plants. One example of such a general rule for connectivity formation would be the genetic definition of a subset of spinal interneurons as commissural interneurons, which allows them to grow their axons across the midline, whereas other spinal interneurons and sensory afferents do not (10). Such ontogenetic specification could, for example, also be important for the topological partitioning of the spinal termination zones (11, 12). We here explore to what extent circuit formation based on learning rather than preprogramming could explain some functional aspects of the spinal cord connectivity patterns observed in adult animals.

Fetal animals generate spontaneous motor twitches that would provide coherences between sensory and motor signals that reflect the mechanics of the musculoskeletal system and could be used for aspects of self-organization (13), for example, pruning of incorrect connectivity and specification of synaptic weights. Specifically, it is well known that the spindle primary afferents (Ia) project monosynaptically and predominantly to the homonymous αMNs that innervate the extrafusal muscle fibers, giving rise to the classical tendon-jerk reflex. However, the Ia to αMN connectivity is not confined to homonymous αMNs. Ia afferents can also make monosynaptic connections with the αMNs of other muscles, particularly those that have a related function (14–16). That observation would suggest that learning, perhaps even before birth, can play a role in establishing this connectivity.

Activation of the homonymous αMNs will tend to shorten the muscle and its spindle receptors, reducing and even silencing spindle afferent activity. A Hebbian learning rule based on enhancing synapses in which there are simultaneous pre- and postsynaptic firing (“neurons that fire together wire together”) would then eliminate rather than promote homonymous Ia-MN connectivity. This type of learned connectivity has previously been achieved in a model of the withdrawal reflex that employed reversed Hebbian learning (17); while interesting, this is not consistent with what is known about spinal learning or typical long-term potentiation.

In mature mammals, spindle afferent activity can also be driven by an independently controlled γMN system that causes contractions or stiffening of various intrafusal muscle fibers and thereby increases the excitability of both the primary (Ia) and secondary (II) sensory endings of the muscle spindle (18–20). However, γMNs differentiate and modulate spindles postnatally and later than αMNs and βMNs (21).

It is often forgotten that a substantial percentage of the spinal MNs in all vertebrates (including mammals) are, in fact, βMNs, which innervate both intrafusal and extrafusal muscle fibers [reviewed by Manuel and Zytnicki (22)]. Furthermore, during fetal development, the spinal MNs of a given motor pool and, to a lesser extent, its synergists, generate synchronous spontaneous activity as a result of gap junctions between them (23). We hypothesize that the widespread fusimotor effects of βMNs will be sufficient to overcome the relatively weak extrafusal contractions during early development, resulting in firing patterns in spindle primary afferents and MNs that could support Hebbian organization of the spinal monosynaptic reflex between them. The short duration muscle twitches known to exist early in development (24) would generate reinforcing sensory feedback while avoiding the spindle unloading caused by skeletal motion and muscle shortening.

To test the feasibility of self-organization of sensorimotor function based on musculoskeletal mechanics and behavioral experience, we have designed a model creature with the simplest musculoskeletal system that we thought might exhibit an interesting range of behaviors. Therefore, the model system does not feature a full replication of the macroscopic configuration of an extant biological musculoskeletal plant but is kept to a minimum with two arms and one flexor and extensor attached to each of the two arms. Skeletal linkages with more degrees of freedom can become highly difficult to control, and an attempt to replicate them would risk consuming most of the effort on solving the high-dimensional control problem rather than the more fundamental issues we aimed to address. Our musculoskeletal plant is controlled by spinal neurons using a linear summation model (LSM) (25), which is a simplified version of a previously published implementation (26), together with synaptic plasticity based on the Hebbian-inspired calcium covariance learning rule (27).

In this report, we describe how random twitching activation patterns such as generated in the fetal spinal cord could give rise to naturally occurring connectivity patterns of spindle Ia afferents to MNs, one of the first circuitry formation events during fetal development (28). We discuss how Hebbian self-organization might have accounted to the emergence of such patterns despite blockage of extrafusal muscle transmission during fetal development (13). The model includes other sensors that contribute to interneuronal circuits that develop at later stages; these are the subject of ongoing studies that are outside the scope of this report. This report provides first steps toward identifying how the central nervous system (CNS) might self-organize so as to learn how to get its musculoskeletal system to perform useful tasks.

## METHODS AND MODEL DESIGN

**Figure 1.**
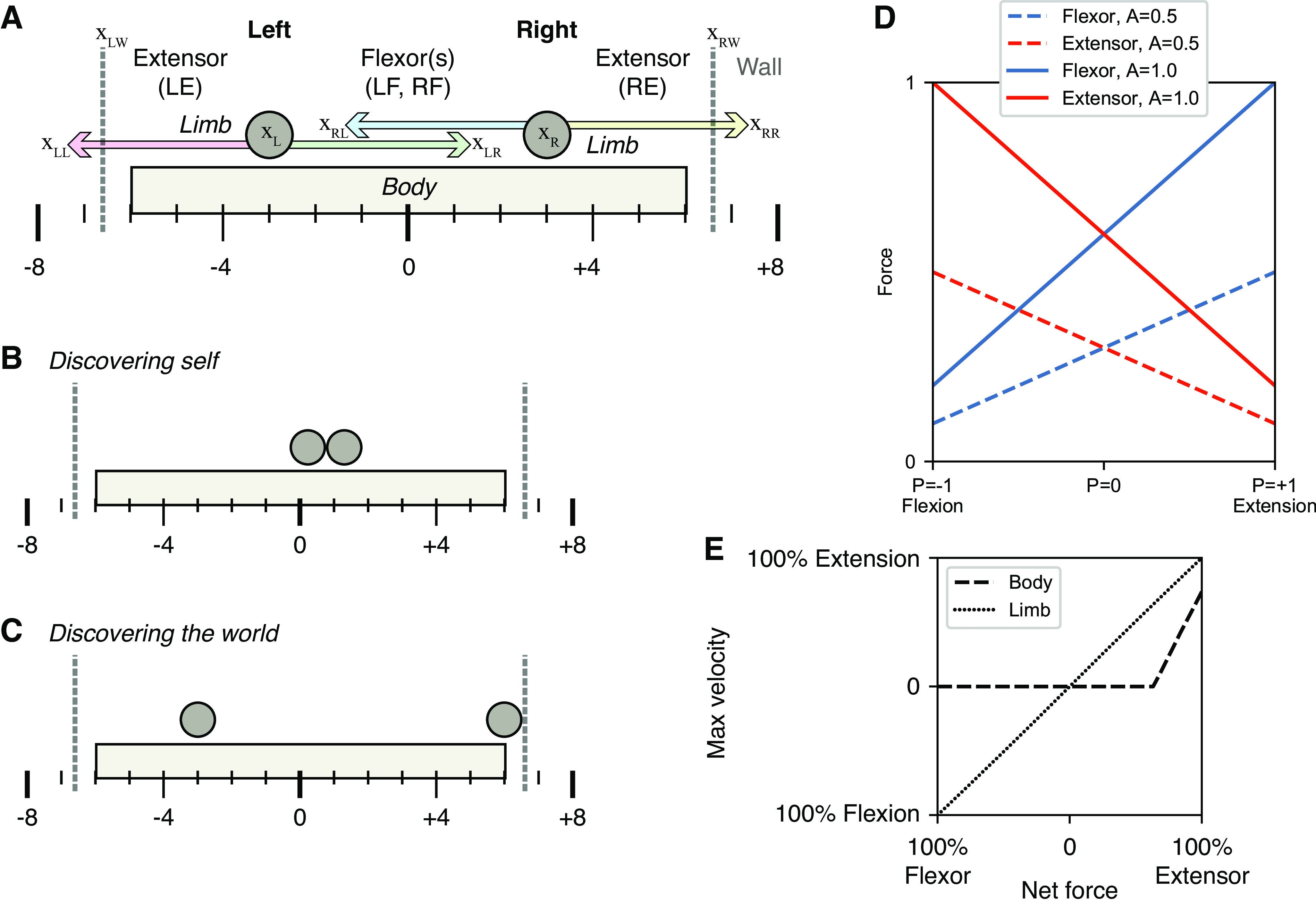
Macroscopic anatomy and behavior of the Oropod model system. *A*: the Oropod anatomy with body, limbs, and corresponding muscles left extensor (LE), left flexor (LF), right flexor (RF), and right extensor (RE). Left and right walls of the Oropod World are indicated with gray dashed vertical lines, and the absolute global positions are indicated below the body; position terms used in *Eqs. 1–4* are pointed out with relative zero at x_LL_. The limbs’ anatomical limits are ±4 units, as shown. *B* and *C:* notable states of the Oropod relevant to later development. *D*: graph of the force output (*y* axis) relative to the position of a limb (*x* axis) and activation of the muscles (line colors according to legend). *E*: graph over max velocity as a function of net force; due to stiction the body cannot move if the net force is not above the threshold.

The model system is depicted schematically in Fig. 1, consisting of the organism (called an Oropod as it only consists of a mouth and feet), a bounded world in which it can move, and one or more objects with their own behavior with which the Oropod can interact (Fig. 1, *A*–*C*). The Oropod is designed to be akin to a one-dimensional cephalopod, with two tentacle-like limbs that can be moved (up to ±4 units along the *x* axis, Fig. 1*A*) so as to contact each other (Fig. 1*B*), to shift the Oropod’s position by pushing against the world boundary (Fig. 1*C*). The Oropod can go through stages of sensorimotor development by first operating in isolation as described in this article, then with added objects with increasing complexity of their autonomous behaviors, and finally by changing its location in the world. The expected behavioral repertoire will be limited to that of submammalian vertebrates, whose anatomy and physiology can provide some useful hints for the design of the Oropod. These later stages are not necessary to test the hypothesis of this report; they enable future hypotheses to be tested within the same gradually developing system.

### Musculoskeletal Mechanics

Each one-dimensional limb is operated by an antagonistic pair of muscles that are unidirectional muscle force-generators (springs that can pull but not push). Extensor muscles pull the limbs outward relative the center of the Oropod body, and flexor muscles pull the limbs inward and toward each other, resulting in a total of four muscles (Fig. 1*A*).

Although large terrestrial organisms have mechanics dominated by inertia and Newton’s second law (force = mass × acceleration), small, aquatic organisms (from which vertebrates evolved) have limb and body mechanics dominated by viscosity, i.e., the force required to move increases with velocity (force = viscosity × velocity; *Eqs. 1* and *2*). The Oropod limbs and body incorporate such viscous damping, allowing us to simplify the muscle model by omitting the force-velocity relationship present in terrestrial organisms. Thus, the velocity of the Oropod’s limb motion is proportional to net force (Fig. 1*E*; net force is the difference in active force generated by the antagonist muscles pulling the limb in opposite directions plus any external force from contact with the end-effector). Furthermore, similar to the biological world, in the Oropod model system the force output of a muscle is a function of both its neural activation and its kinematic condition (i.e., activation multiplied with current muscle length), which gives rise to “preflex” responses to perturbations (29). The Oropod muscles incorporate the well-known “spring-like” property of muscles operating on the ascending limb of their force-length curve (Fig. 1*D*). The spring curves have a midrange overlap so that the organism can control “stiffness” by using different levels of co-contraction of antagonists.

(*1*) $$B\dot{x}=F_{L}=-A_{\mathrm{LE}}x_{L}+A_{\mathrm{LF}}\left(x_{\mathrm{LR}}-x_{L}\right)-{\dot{x}}_{L}D-F_{C}\text{, (force left limb),}$$

(*2*) $$B\dot{x}=F_{R}=-A_{\mathrm{RF}}\left(x_{R}-x_{\mathrm{RL}}\right)+A_{\mathrm{RE}}\left(x_{\mathrm{RR}}-x_{R}\right)-{\dot{x}}_{R}D-F_{C}\text{, (force right limb),}$$

(*3*) $$F_{\mathrm{LW}}=A_{\mathrm{LE}}\left(L_{\max}-x_{\mathrm{LW}}-x_{L}\right)-A_{\mathrm{LF}}(x_{\mathrm{LR}}-x_{L}),\text{(force left wall, valid for positive forces),}$$

(*4*) $$F_{\mathrm{RW}}=-A_{\mathrm{RE}}\left(L_{\max}-x_{\mathrm{RW}}-x_{R}\right)+A_{\mathrm{RF}}(x_{\mathrm{RR}}-x_{R}),\text{ (force right wall, valid for negative forces),}$$

(*5*) $$F_{\mathrm{Body}}=F_{\mathrm{LW}}-F_{\mathrm{RW}}-\dot{x}D\text{,}(\text{force body valid for positive }F_{\mathrm{LW}}\text{ and negative }F_{\mathrm{RW}})\text{,}$$

In the earlier equations describing the force relationships, *B* denotes the viscosity, *x* is position relative most leftward possible limb position, *A* is muscle activation, *D* is damping due to viscosity, and *L*_max_ is maximal muscle length. Subscript L and R denote left and right, respectively; subscript LW and RW denote left and right wall, respectively; and subscript LL, LR, RL, RR denote Left limb Left muscle, Left limb Right muscle, Right limb Left muscle, and Right limb Right muscle, respectively. Refer to Table 1 for a complete list of all variables used in this paper. Damping is a constant term due to our assumption that we model an organism dominated by viscosity and is set to 2.0 and thus renders the limb movements overdamped. Finally, *F*_C_ is external contextual forces, which will be mostly due to contact with limbs, walls, prey, and anatomical stops.

**Table 1.** Parameter definitions

Parameter	Symbol	Value or Range
Position along the *x* axis relative a zero-point at the far left	*x*	−inf to inf
Damping	*D*	2.0
Muscle length	*L*	0 to 1
Muscle activation	*A*	0 to 1
Neuronal activation (membrane potential). Superscript plus denotes the positive part of the range.	*P*	−1 to 1
Sign matrix denoting excitatory or inhibitory synapses	*S*	+1 or −1
Synaptic weight. Superscript plus denotes the positive part of the range, which is the functional part of the range.	*w*	−1 to 1
Learning signal	*l*	−1 to 1
Compensation factor	*c*	0 to 1
Learning rate	*η*	0 to 0.001
Kalman gain for activity	*K*_A_	0.3
Kalman gain for learning signal	*K*_L_	0.001
Kalman gain for mean activity	*K*_M_	4.0e-5
Filter function for synaptic activity. High-pass (0.05 Hz threshold) for EPSP and low-pass (0.2 Hz threshold) for IPSP.	φ_L_	Function
One-dimensional Kalman filter	φ_K_	Function

The Oropod body also has stiction, represented as a threshold value of net force before the body starts to move, which avoids accidental drifts in location in its world as a result of cumulative effects of accelerating the small inertial masses that are required to avoid singularities in most physics engines. This stiction has been set fairly high (∼50% of maximal muscle force, Fig. 1*E*) so that the Oropod maintains its current position even while pushing against the world with low forces.

As our aim was to model dynamics in the fetal stage when muscle strength is reduced compared to the adult stage (30), we had to define muscle strength as a feature that could change with maturation. Accordingly, we defined full muscle strength to be when the organism could move one limb from one extreme position (full flexion/extension) to the other in a short but not unreasonable time (Fig. 4*A*). This was defined as roughly 2 s from an animation of the model, and the muscle strength at this point was defined as 100% muscle force. For the main simulations of the organism, the muscle strength was reduced to 10% to model the decreased strength during the fetal stage.

In summary, the Oropod has limb and body dynamics dominated by velocity and muscle dynamics dominated by length (limb position), whereas real terrestrial organisms have limb dynamics dominated by acceleration and muscle dynamics dominated by length and velocity (31).

### Somatosensation

**Figure 2.**
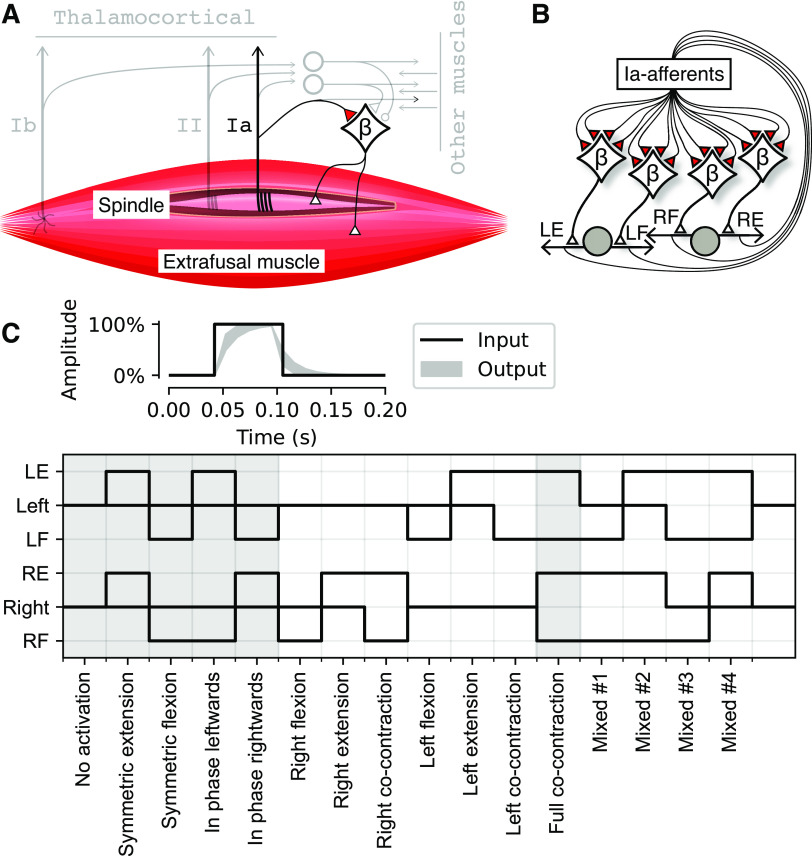
Muscle and neural network properties of the Oropod. *A*: proprioceptive receptors of the Oropod muscle. In this report, we focus exclusively on the Ia-MN synapse indicated in red; other afferent and interneuronal elements are depicted in gray. *B*: illustration of the neural network design of the Oropod. The Ia afferent from each muscle synapses initially onto the βMNs controlling all four muscles (both extra- and intrafusal muscle fibers). Limbs and muscles are drawn as in Fig. 1, *A*–*C*. *C*: in the top is an example of a twitch APG with 100% amplitude and 50-ms duration; the black line shows the internal square pulse into the APG and the light gray area shows the output range when fed through the one-dimensional Kalman filter with the gain range 0.5–0.8. It is the output that is injected into the MN synapse. Below are all 16 possible binary permutations of the phasic APG output and hence βMN activation (but note that in the actual simulations both the amplitudes and unitary durations were also varied). Name of each permutation is indicated along the *x* axis. Gray backgrounds indicate permutations used to generate Figs. 5 and 7. APG, activity pattern generator; Ia, primary afferent; MN, motoneuron.

**Figure 3.**
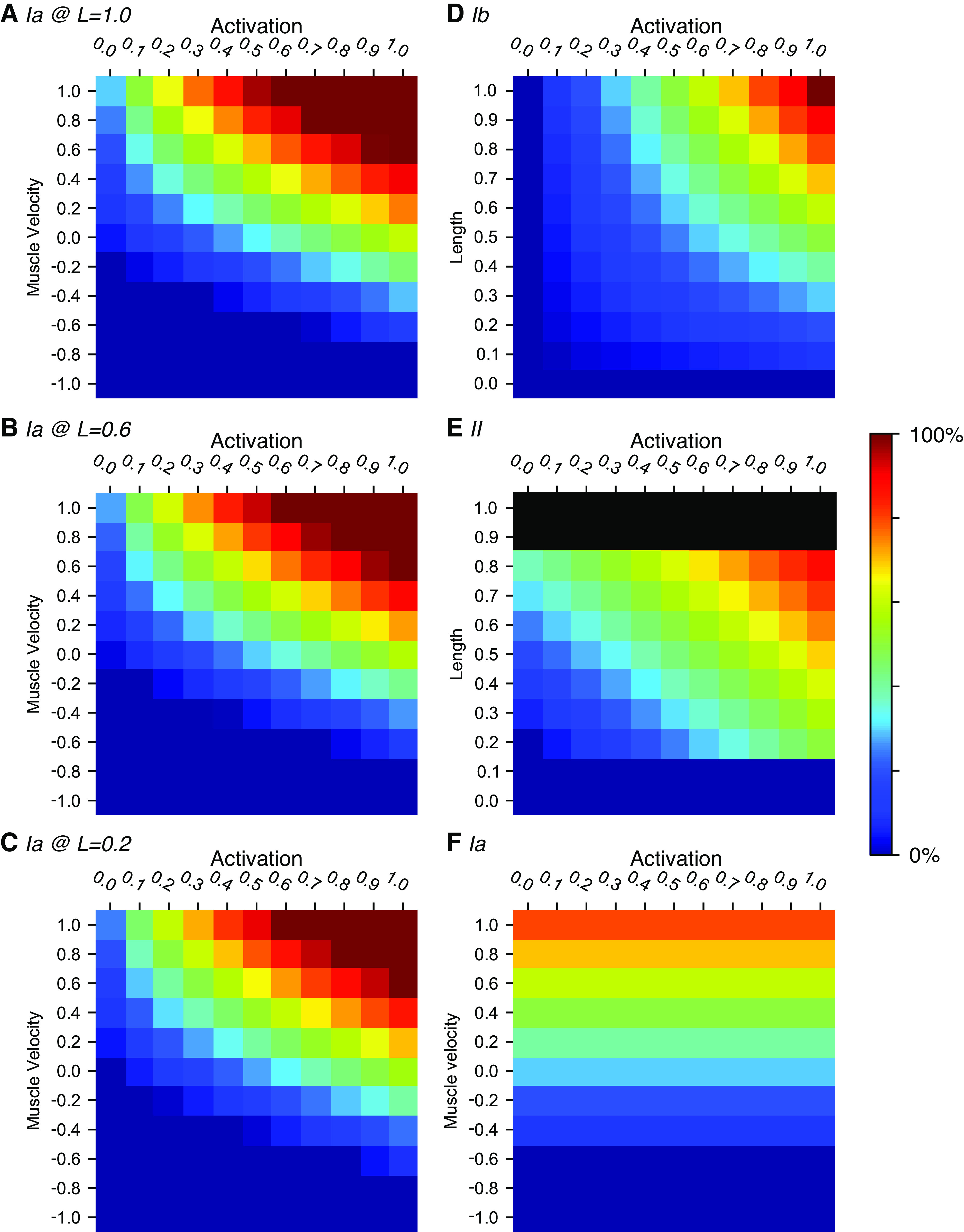
Output heatmaps illustrating the tuning of the sensors. *A–C*: group Ia sensor output with muscle lengthening speed along the *y* axis, and MN muscle activation along the *x* axis. The II term of the Ia sensor activation (as defined in *Eq. 6*) varies with muscle activation, whereas muscle length is set at 1.0 in *A*, 0.6 in *B*, and 0.2 in *C*. Muscle length 0.6 corresponds to the neutral position and 0.2 to the minimum length possible. *D*: group Ib sensor output with muscle length along the *y* axis and MN muscle activation along the *x* axis. *E*: group II sensor output with muscle length along the y-axis and MN muscle activation along the *x* axis. Note that the minimum muscle length possible is 0.2. *F*: group Ia sensor output without fusimotor effect, thus only coding for muscle velocity and length. The muscle length is set to 0.6 in this heatmap. MN, motoneuron.

Each Oropod muscle has proprioceptors that encode velocity, length, and force generation (Fig. 2*A*), consisting of group Ia muscle spindle afferents, group II muscle spindle afferents, and Golgi tendon organs (Ib), respectively. The response dynamics of these sensors during a predefined set of muscle activations (without any connected neural network) are illustrated in Fig. 5. Our model ignores other mechanoreceptors in muscles, joints and skin that contribute to proprioception. The Oropod Ib receptor is modeled as a simple, linear sensor of active force being generated by the muscle in which it resides (Fig. 3*D*) (32, 33). The spindle receptors (Ia and II) are discussed in more detail in the sections *The Type II Muscle Spindle Afferent* and *The Type Ia Muscle Spindle Afferent*. The present study covers an early stage of fetal development in which only the Ia sensor has functional projections to the spinal circuitry (34).

### Beta Motoneurons for Fusimotor Drive

As noted in the introduction, the natural tendency of extrafusal muscle fibers to shorten and thereby silence the spindle stretch receptors argues against the feasibility of Hebbian learning as a basis for the homonymous monosynaptic stretch reflex. However, amphibian and fish muscles are generally innervated by βMNs that simultaneously activate extrafusal and intrafusal muscle fibers (35). Mammalian muscles also generally have a substantial but variable percentage of βMNs alongside their much more evolved and independent αMN and γMN subsystems that provide independent control of extrafusal and intrafusal muscles fibers, respectively (22). The fusimotor effects of βMNs are likely to be weak in adult mammals. βMNs probably fire at the same 10–50 pps as αMNs (36) whereas γMNs generally fire at 50–200 pps [reviewed by Ellaway et al. (37)]. During early fetal development, however, extrafusal muscle fibers are immature and weak (30), γMN activity may be absent or uncoordinated (21), and the body tends to be confined in utero or in ovo. In this situation, the fusimotor effects of activating a βMN would likely produce a net increase in firing of the afferents in the spindle that it innervates. Thus, the βMNs could potentially support a learning-based organization of the Ia-MN connectivity. All or most αMNs and βMNs in a given motor nucleus are likely to be corecruited as a result of fetal tight junctions among them (38).

### Spindle Receptors and Fusimotor Function

It is important to consider the complexities of spindle receptor and fusimotor function that are largely preserved across vertebrate phylogeny (39, 40). There are at least two distinct types of intrafusal muscle fibers, which have different mechanical effects on the sensory receptors that wrap around them. The group II receptor wraps around and detects simple length changes in fast-twitch intrafusal fibers (chain and bag2 types) whose fast-twitch contractions when activated can overcome shortening produced by extrafusal muscle, resulting in increased group II firing at a given muscle length. The Ia receptor is wrapped around all of the intrafusal fibers, but its location on the bag2 fiber is in the middle region where the bag of nuclei displaces myofilaments. Active contraction of the bag2 poles results in much larger biasing effects on the Ia than II receptor. The Ia receptor alone wraps around the compliant middle region of the bag1 fiber, a slow muscle type that becomes increasingly viscous at the poles with activation but does not shorten appreciably. This results in a mechanical high-pass filter for externally applied length changes that accounts for activation-dependent velocity sensitivity of the Ia. These two types of Ia modulation tend to kick in at different levels of extrafusal recruitment. When mammals want to execute large, rapid movements, they recruit static βMNs that activate “fast” muscle fibers both extrafusally (fast-twitch = type II) and intrafusally (bag2 and chain). The intrafusal contractions result in “static” increases in activity of both spindle Ia and II afferents, essentially a positive bias that will keep these receptors from being completely unloaded and silenced during the large, rapid shortening of the whole muscle that is expected from the strong extrafusal muscle contraction. When mammals want to use muscles only to stabilize existing postures, they recruit only the “dynamic” subset of their βMNs that innervate slow (nontwitch) intrafusal muscle fibers (bag1) that enhance velocity sensitivity and slow and weak extrafusal fibers (actually slow-twitch or type I in mammals) (41). Finally, adult mammals are equipped with two types of γMN (static and dynamic) that the CNS activates independently of the α and βMNs to provide differential control of length and velocity sensitivity of spindle afferents (18, 40); γMNs differentiate postnatally (21) and their effects neonatally are unknown.

We have modeled the Oropod muscle spindles as being all-β innervated, like in amphibian systems whose anatomy but not physiology is well described, while using the well-described and modeled physiology of the mammalian spindle to combine both static and dynamic fusimotor effects.

All somatosensory afferents generate excitatory postsynaptic effects centrally that are scaled and clipped, if necessary, to have a dynamic range that is 0 to +1. The ranges of possible lengths and velocities of the Oropod muscle are constrained by its limb mechanics, so the internal coordinates for each muscle can be normalized such that each muscle length (*L*) has the range 0 to +1 and each muscle velocity (*V*) has a range from −1 to +1 (42). Muscle activations (*A*) and all sensory afferents range from 0 to +1.

Assuming the earlier normalizations, we define the sensitivity of the Oropod spindle receptors in the following sections.

### The Type II Muscle Spindle Afferent

Spindle secondary (II) afferents do not project directly to MNs in reality or in our model, but their transduction mechanisms represent a subset of those for the Ia afferents (19). The length-dependent output of the II afferents is defined in *Eq. 6* (and visualized in Fig. 3*E*), which constitutes one term of the Ia sensitivity by virtue of their common receptor endings on the chain and bag2 intrafusal fibers. The *A* term provides the static fusimotor effect that scales linearly with extrafusal activation. Thus, the II sensor would reach its maximal value of +1 only when the muscle is maximally stretched and maximally activated, e.g., during the reversal of a maximal waving motion.

(*6*) $$II=\frac{\left(L-0.2\right)\times 1.25+A}{2.0}\text{, (spindle II).}$$

### The Type Ia Muscle Spindle Afferent

Spindle primary (Ia) afferent output is defined in *Eq. 7* and visualized in Fig. 3, *A*–*C*. Its muscle velocity sensitivity depends nonlinearly on muscle activation so that it increases rapidly for lightly recruited muscle and then saturates, simulating the early recruitment of the dynamic βMNs. Its length sensitivity is similar to the spindle II but with an added activation-dependent term reflecting the strong effect of wrapping around the middle region of the bag2 intrafusal muscle fiber.

(*7*) $$Ia=\frac{\left(1.5+log_{10}\left(A+0.1\right)\right)\times V+A+\left(II\times 0.2\right)}{2}\text{, (spindle Ia).}$$

The maximal velocity of stretch of an Oropod muscle occurs when it is passive at its shortest length (*A* = 0, *II* = 0) and its maximally activated antagonist is at its longest length. This will generate output Ia = +0.25. In a maximally activated muscle (*A* = 1), maximal velocity of stretch (*V* = +1) would saturate Ia at max (which is clipped by the range of 0–1). That condition can never arise without external interference because turning the muscle on necessarily decreases its rate of stretch by the antagonist. A maximally active muscle that is shortening at maximal muscle velocity (*V* = −1) would saturate at Ia = 0 (which is physiological in mammals). A muscle that is active at *A* ≈ 0.22 could shorten at maximal *V* ≈ −0.22, which would result in output Ia = 0. Further increase in muscle activation or obstruction of shortening from antagonist or external load would result in a strong velocity signal (which is physiological in mammals).

For our control experiments, we also created a sensor devoid of all fusimotor effect, simulating a context with only αMNs (*Eq. 8*; visualized in Fig. 3*F*).

(*8*) $$\mathrm{Ia}=\frac{L+V}{2}\text{, (spindle Ia without fusimotor effect).}$$

### Neuronal Model Design

To evaluate our hypothesis of self-organization of sensorimotor function based on musculoskeletal mechanics and behavioral experience, we needed an artificial neuronal model suited for repeated simulations of arbitrary neural network (NN) connectivity among a variety of afferent and interneuron types. To this end, we used a linear summation neuron model with dynamic leak (25) together with learning rules similar to previous simulations of cuneate neurons (26), i.e., Hebbian-inspired calcium co-variance learning rule.

### Network Connectivity

Each of the four Oropod muscles is controlled by a single MN and has three proprioceptors that project to the NN (Fig. 2*A*). In this report, however, we are concerned only with the Ia primary afferents that project directly to all four MNs (Fig. 2*B*), which II and Ib do not.

Muscles are not commonly controlled by a single MN alone. Rather, motor pools made up of hundreds of MNs controling the activation of each muscle, thus enabling the muscle to be gradually activated by cumulative recruitment of motor units. In early development, the activities of these MNs are synchronized and proportionally recruited by means of tight junction coupling (38). Based on this observation, and for purposes of model simplification, we here reduced the motor pool of each muscle into a single MN with a scalar output (*P^+^,* defined in *Eq. 10*) with the functional range 0–1. However, due to the nature of the neuron model the hyperpolarized state of each neuron depends on the presence of inhibitory neurons. In this paper we do not have any inhibitory neurons and therefore the MNs would only be in their dynamic active range. To compensate for this, we added a small hyperpolarizing bias to the output activity from each MN resulting in that the muscle activation (*A*) was defined as positive-only:

(*9*) $$A={\left(\frac{P^{+}-0.1}{0.9}\right)}^{+}\text{, }(\text{muscle activation})\text{.}$$

### Neuron Model

Physiologically, our neuron model is electrotonically compact in the sense that the synaptic voltage signals are added linearly to the somatic voltage. In our model we have two categories of “biochemical” compartments, a main, somatodendritic compartment and synaptic compartments, where the compartmentalization serves the sole purpose of controlling the learning. This approach has been used to model learning in cuneate neurons (26). Cuneate neurons, however, have relatively complex calcium dynamics in their main compartment that the synaptic inputs can trigger. Here, we simplified that model to instead make the calcium concentration (as a dimensionless quantity) of the main compartment equivalent to its voltage (which in turn was defined by a linear summation of the synaptic inputs).

### Neuronal Compartment Model

The output of most CNS neurons is the action potential. However, in our previously published neuron model spiking is omitted, and each output signal of a neuron is instead a time-continuous, positive-only voltage signal (*P^+^*), which can be thought of as representing the combined output of a population of asynchronously firing neurons of a given type (25). This simplification is valid because the spike output of the neuron reflects a somewhat noisy probability density function of its membrane potential (43, 44). The discreteness associated with the timing of single spikes in individual sensory neurons can be assumed to be mostly averaged out across the population of asynchronously firing neurons of the same type (45).

Signal integration in neurons depends on the establishment and the modulation of the membrane potential. The resting membrane potential depends on the background leak conductance, which is dominated by potassium. Modulations of this membrane potential are generated by the activation of chemically or electrically gated conductances for specific ions (i.e., in excitatory synapses the chemically gated conductances are typically dominated by sodium). Action potentials are generated with a frequency that is more or less linearly related to the degree of depolarization (44).

Background excitatory or inhibitory synaptic activity itself reduces the response to any particular synaptic input because it is accompanied by opening conductance channels in the postsynaptic membrane (46–49) and thus results in shunting. This is particularly important in a developmental model of the NN in which the number and strength of synaptic inputs onto any given neuron is expected to undergo large changes [see also Tsianos et al. (50)].

Rather than simulating the conductances explicitly, these effects are instead emulated using *Eq. 10*, where $P_{i}^{+}$ is the positive output potential from the *i*th neuron, which depends on the input from *j*th neuron ($P_{j}^{+}$), modulated by the positive synaptic connection weight ($w_{ij}^{+}$) and a sign matrix where excitatory synapses are positive and inhibitory are negative (*S_ij_*). The shunting is represented by the sum of synaptic activation plus a resting leak constant (*k_i_*) in the denominator which is twice the rolling sum (with a lower bound of 0.5) of synaptic activation (*x*) as obtained by *Eq. 11* (*K* is the gain factor). The summed synaptic activity in both the numerator and in the denominator are low-passed filtered using a Kalman filter as described in *Eq. 11*.

(*10*) $$P_{i}=\frac{\varphi_{K}\left(\sum P_{j}^{+}w_{ij}^{+}S_{ij},\;K_{A}\right)}{k_{i}+\varphi_{K}\left(\sum P_{j}^{+}w_{ij}^{+},\;K_{A}\right)},\text{ (neuronal activation),}$$

(*11*) $$\varphi_{K}(x,\;K)=y_{t}=y_{t-1}\left(1-K\right)+x_{t}K\text{, (Kalman filter),}$$

(*12*) $${\bar{P}}_{i}=\varphi_{K}(P_{i}^{+},\;K_{M})\text{, (mean activity).}$$

This neuronal activation behavior has the important feature of protecting each neuron from saturation during development because each active synapse adds to the activation with a potentially increasing weight while adding increasingly to the leak.

*Equation 10* provide a complete description of how information flows through our neuronal model. The output from any network configuration composed of these neurons depends on positive-only weights of both excitatory and inhibitory synapses, modulated by total synaptic activity.

### Synaptic Weights

The synaptic weights in our model are scalars with a functional range of 0 to +1 for the excitatory Ia-MN synapses studied here. There is no information on fetal synaptic weights, and thus we made the assumption that synapses have randomized initial weights at the low end of the spectrum. In our model the initial synaptic weights are random samples from a normal distribution (mean µ = 0.2, standard deviation σ = 0.16, synaptic weights below 0.001 are reseeded thus rendering all initial weights positive). The model can in theory reduce the synaptic weights into the negative range. This can only happen through learning and would have the effect that the synapse would be nonfunctional since it is only the positive part that is used in equations. Inhibitory effects are not controlled by the sign of the weight; inhibition and excitation are fixed according to the static sign matrix (S).

### The Dynamics of the Learning Mechanism

During the course of a simulation, the synaptic weights are continuously adjusted according to the correlation between the activity of the individual synapses and the activity level of the neuron as a whole. This learning mechanism is in essence the calcium co-variance rule (27, 51) implemented such that both Hebbian LTP and LTD of synaptic plasticity can occur. The core learning rule is an adaptation of Oja’s rule (52) and is described in *Eq. 13*. The main difference between this equation and Oja’s rule is that instead of a binary parameter that allows for update or not, a scalar learning signal parameter has replaced it (denoted *l*, *Eq. 14*, subscript *ij* indicates the *j*th synapse of the ith neuron). The dynamic that governs the learning signal has several important aspects, but essentially the degree of correlation between the activity of the synaptic compartment and the neuronal compartment (*Eq. 14*) is what will define the fate of the synaptic weight. This learning signal is a scalar parameter in the range of −1 to 1, and thus allows for scalable potentiation and depression of the synapse in question. Depending on the sign of the learning signal, and thus the direction of update, the compensation factor (*c*) is changed according to *Eq. 15*. Finally, a learning rate parameter (*η_i_* for the ith neuron, *Eq. 16*) scales learning.

(*13*) $$\Delta w_{ij}=l_{ij}\eta_{i}c_{ij}\text{, (learning rule),}$$

(*14*) $$l_{ij}=\varphi_{K}(P_{j}^{+}w_{ij}\left(\varphi_{L}\left(P_{i}\right)-{\bar{P}}_{i}\right),\;K_{L})\text{, (learning signal),}$$

(*15*) $$c_{ij}=\{\begin{array}{c} 1-w_{ij},\;l_{ij}\geq 0\\ w_{ij},\;l_{ij}<0 \end{array}\text{, (compensation factor),}$$

(*16*) $$\eta_{i}={\bar{P}}_{i}^{4}\times 0.01\text{, }(\text{learning rate})\text{.}$$

### Learning Signal

The mean potential ($\bar{P}$ in *Eqs. 14* and *16*) represents several more complex features of the internal machinery of the neuron. First and foremost it is intended to model the current balance of phosphatases and kinases in the neuron (53), which is assumed to be controlled by the total calcium level, which in the present model is simply the output activity generated by the neuron over time. A higher concentration of kinases, for example, can make the probability for LTP higher, whereas a higher concentration of phosphatases would instead increase the probability for LTD. A high probability of LTP will over time result in a higher output activity of the neuron, in which case our model neuron would respond by shifting the balance towards phosphatases to prevent overexcitation. An ongoing adaptation of this polarity threshold hence helps preventing saturated learning in the neuron (26). In the current system with the synaptic input from the sensors being spontaneously active, it has the net effect of implementing homeostatic synaptic plasticity, or synaptic scaling (54). A slight high-pass filter ($\varphi_{L}$) was applied to the EPSP (discrete neuronal activation, *P*) to emulate the faster supralinear calcium signal that is generated under excitatory synaptic activation (26).

### Autonomous Activity Pattern Generator

In any organism or model thereof, a central issue is how activity initially arises and how it is subsequently molded into increasingly useful behavior. In the mature spinal cord the notion of a central pattern generator (CPG) is ubiquitous [for a review, see Guertin (55)]. In many envisaged instantiations, the CPG has the power to produce functionally synergistic contractions of muscles, and thus produce movements that are functional, hence evolutionarily sound. However, it is still not clear how or when the formation of the CPG would reach such a stage of maturation. Here we chose the more assumption-free approach of letting the MNs generate their own excitation internally. This approach seems to be supported by evidence for pacemaker potentials in MNs arising from a high level of voltage-gated calcium channel expression and resultant calcium positive feedback responses in early developmental stages (24, 56). These and other intrinsic conductances support the generation of pacemaker-like potentials in individual MNs. Given the high prevalence of gap junctions among the MNs of a given motor pool early in development (38, 57), such pacemaker activity output could recruit large numbers of MNs synchronously and proportionally, which would account for visible limb twitches in fetal animals. In our model system, such autonomous activity pattern generation is assumed to synchronize across the MNs of each muscle, so we have modeled only one composite MN for each muscle. Our twitch activity pattern generator (APG) represents the internally generated pacemaker potentials initiated at random in the single MN pools, which activate the MNs of separate muscles independently in random patterns.

### APG Patterns Generated in the Simulations

Each twitch APG pacemaker potential has four parameters: amplitude, duration, rise time, and probability of activation. Each parameter was set to avoid biasing the model toward any particular solution in the absence of systematic experimental data about these parameters.

The amplitude of activation was assigned a random value between 0 and 1 from a uniform distribution. As we employed the simplification of modeling the entire MN pool as a single neuron, we had to let that particular neuron be able to express the full range of activation amplitudes.

**Figure 4.**
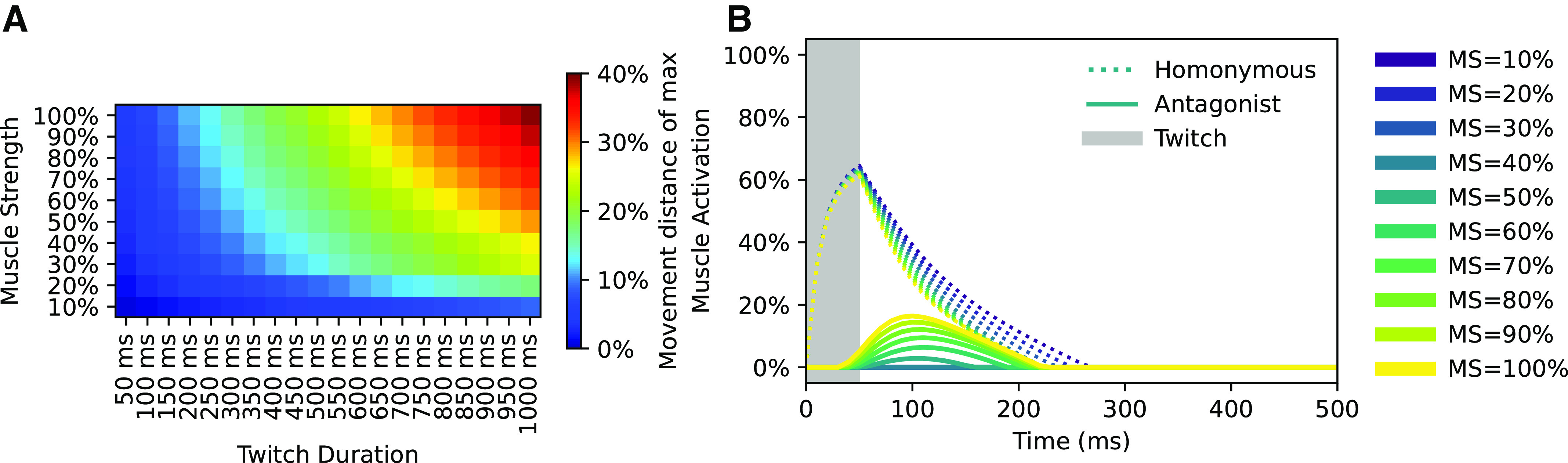
*A*: relation between twitch duration (*x* axis) and muscle strength (*y* axis) with respect to kinematic response. Kinematic response (100%) has been defined as movement from neutral position to the full anatomical stop in one direction. *B*: muscle activation responses due to a 50 ms 100% amplitude twitch into the homonymous MN with a connected NN with 100% synaptic weights for the homonymous Ia-MN synapses and 0% for the remaining, for 10 steps of muscle strength indicated by the color scale at right. A response from the antagonistic muscle is seen when the muscle strength increases above 50% after training of the Ia-MN synapses. MN, motoneuron; MS, muscle strength; NN, neural network.

The duration of each twitch APG potential was used to mimic the observed short muscle twitches in the embryological stage (24). For the primary set of simulations, the duration of each APG potential was randomly assigned a value between 50 and 100 ms from a uniform distribution. The kinematic effect on the homonymous muscle of varying twitch amplitudes and durations can be seen in Fig. 4*A*. After maturation of muscle strength and learning in the Ia-MN synapses, even the shortest duration twitches (50 ms) produce limb movements that are large enough to produce a reflexive response from the antagonist muscle (see Fig. 4*B* and results).

**Figure 5.**
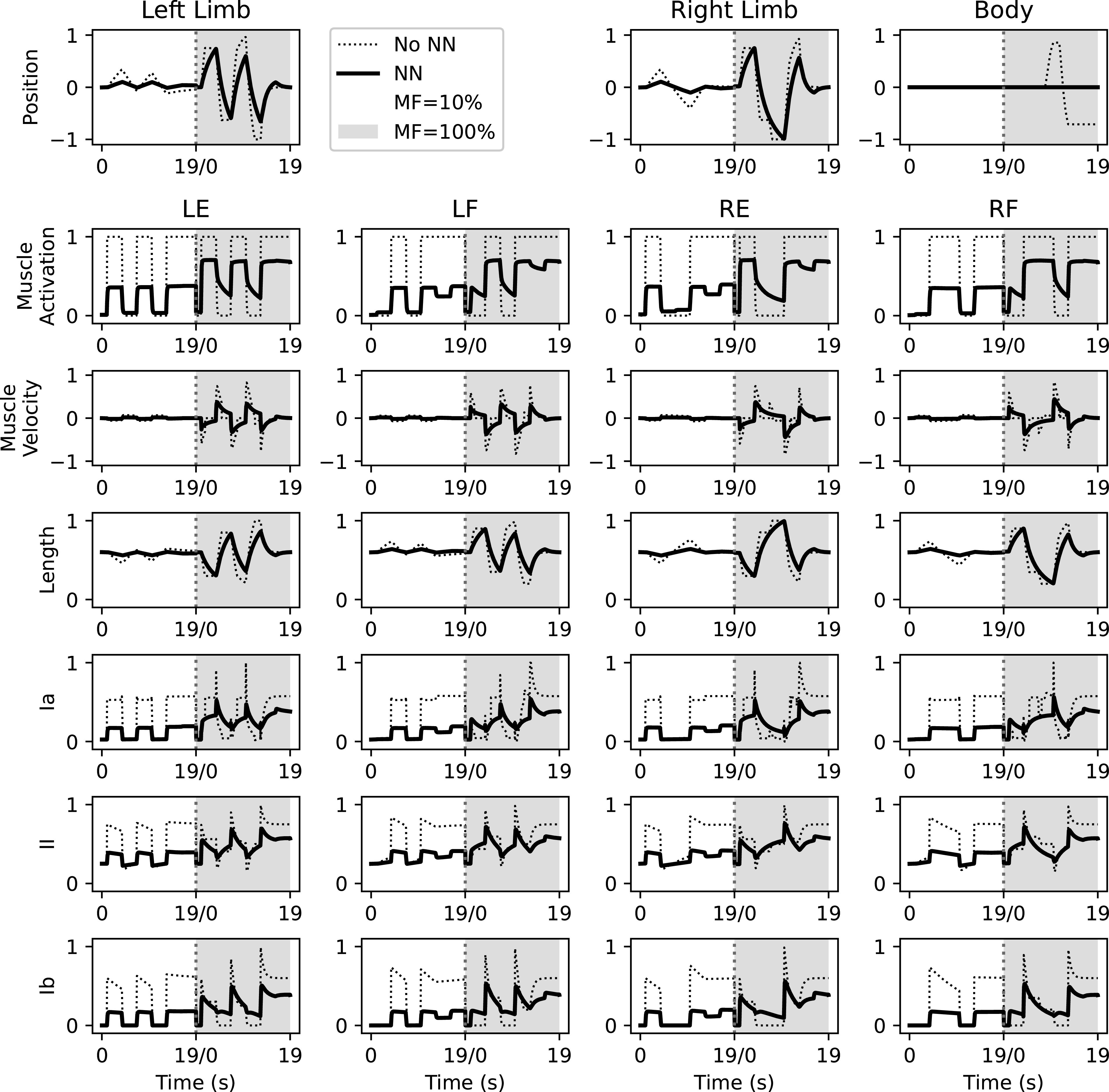
Plant dynamics with a duration of 19 s for a predefined set of muscle activations for selected phasic APG patterns identified in Fig. 2*C*. Dotted lines indicate plant dynamics without any connected neural network. Solid lines indicate plant dynamics with a connected neural network. During the first 19 s (white background) the muscle strength of the plant is scaled down to 10% of max, and the second iteration (gray background) the muscle strength is set to 100% of max. During 10% muscle strength context the synaptic weights were at their initially randomized weights; during the 100% muscle strength context the synaptic weights correspond to the mean end weights of Fig. 6*A*. The first row plots relative position of respective limb and body with respect to their neutral position. Subsequent rows show individual muscles activations, kinematics and proprioceptive activity as labeled. The reason for the decline in all response amplitudes when a neural network has been connected is the leaky integration seen in *Eq. 10*. APG, activity pattern generator.

To obtain more physiological behavior, we also applied a one-dimensional Kalman filter to each of the generated APG potentials with a gain randomly assigned between 0.5 and 0.8 from a uniform distribution. This had the effect of smoothing the otherwise square-pulse inputs (Fig. 2*C*).

Finally, the probability of activation governs the frequency of muscle twitches. This feature of the twitch APG potentials will have two main impacts to be noted. First, since the generators of APG potentials for each MN are independent, an increase in probability of activation would increase the probability of two or more muscles to be active at the same time. This is important because the existence of four muscles results in 16 different permutations of activation (Fig. 2*C*), each of which has the potential to generate different plant dynamics in different contexts. For example, strong extensor muscle activity starting from a neutral limb position results in very different dynamics as opposed to starting from an extreme end-position. In a more complex plant this would include co-activation of synergists. Because each twitch APG potential is independent, however, an increased overall probability of activation increases the probability for co-activation of antagonist or contralateral muscles. In the event of consistent co-activation, a Hebbian learning rule would strengthen the synaptic projections between sensory feedback and all active MNs resulting in a nonfunctional connectivity. This potential problem of spurious connectivity will be less prevalent in a plant with more muscles because the probability of repeated, specific co-activation would decrease. Previous work has touched briefly on this topic [probability of activation set to 11% in a study by Petersson et al. (17)], but we here explore it further.

We also created various phasic APGs that generated longer lasting activation of specific combinations of muscles such as might be generated by a central pattern generator (CPG) composed of genetically hardwired interneurons [as suggested by Lundberg (58) and Grillner (59)]. These variants include *1*) co-contractive activations are prohibited, *2*) only symmetrical movements are allowed, and *3*) only in-phase movements are allowed. Some of these phasic patterns tend to result in limb movements that are impeded or obstructed by contact between limbs or by contacts between a limb and a wall.

### APG Implementation

In the current model system, the twitch/phasic APG was implemented as an independent excitatory synapse on each MN. The weight of that particular synapse was set to the max allowed weight (1.0) and was not allowed to learn. The output from the APG as described in *Autonomous Activity Pattern Generator* was projected into this synapse. The following integration into the overall activity of the particular MN followed the rules outlined in *Neuronal Compartment Model*.

### Simulation

The nervous systems of real organisms are closed loops where the output affects the plant in some way, and these effects together with changes in the physical world create the sensory feedback that is projected back into the nervous system. The continuous sensory feedback is then mixed with internal signals and produces an updated output from the nervous system. The emerging patterns of presynaptic and postsynaptic activity drive the Hebbian learning rule, which continuously changes the gains of this sensory feedback. We emulated this loop with our artificial plant (see initial part of methods and model design), our artificial nervous system (see *Neuronal Model Design* for details), and an artificial physical world as follows:

1.  The state of the Oropod plant and its interaction with the surrounding physical world was continuously updated by the physics engine (pymunk, http://www.pymunk.org/) using an integration time step of 10 ms.
2.  The contractile force generated by each muscle in the plant was updated according to its current length and the output from each corresponding MN, thus updating the state of the plant.
3.  The time evolving activity of the Ia sensors and APG was integrated in each MN as described in the section *Neuronal Compartment Model*, thereby updating the output from each MN and adjusting synaptic weights accordingly.

We saved the state of the NN every 500 s throughout our simulations. This allowed us to load any saved state and run analysis on that particular state. Each simulation ran for 20,000 s, which was a duration that allowed synapses that were initially set to a very low value to adapt to a stable higher value.

We could load a NN state into the loop described above and let it perform a specified set of movements. This was achieved by replacing the twitch APG with the phasic APG that produced a fixed set of muscle combinations (defining which MNs to activate also with respect to amplitudes and durations; Fig. 5).

## RESULTS

We have designed a model creature with a simple set of musculoskeletal mechanics and biologically derived sensor tunings (Figs. 1 and 3). The model creature was controlled by a neuronal network with biologically derived synaptic learning rules and a simple, randomized activity generator in order to test the hypothesis that predominantly homonymous projections from spindle primary afferents to motoneurons as exhibited by the adult spinal cord could result from learning-driven self-organization (Fig. 2).

### Random MN Activations Promote Spinal Connection Patterns

**Figure 6.**
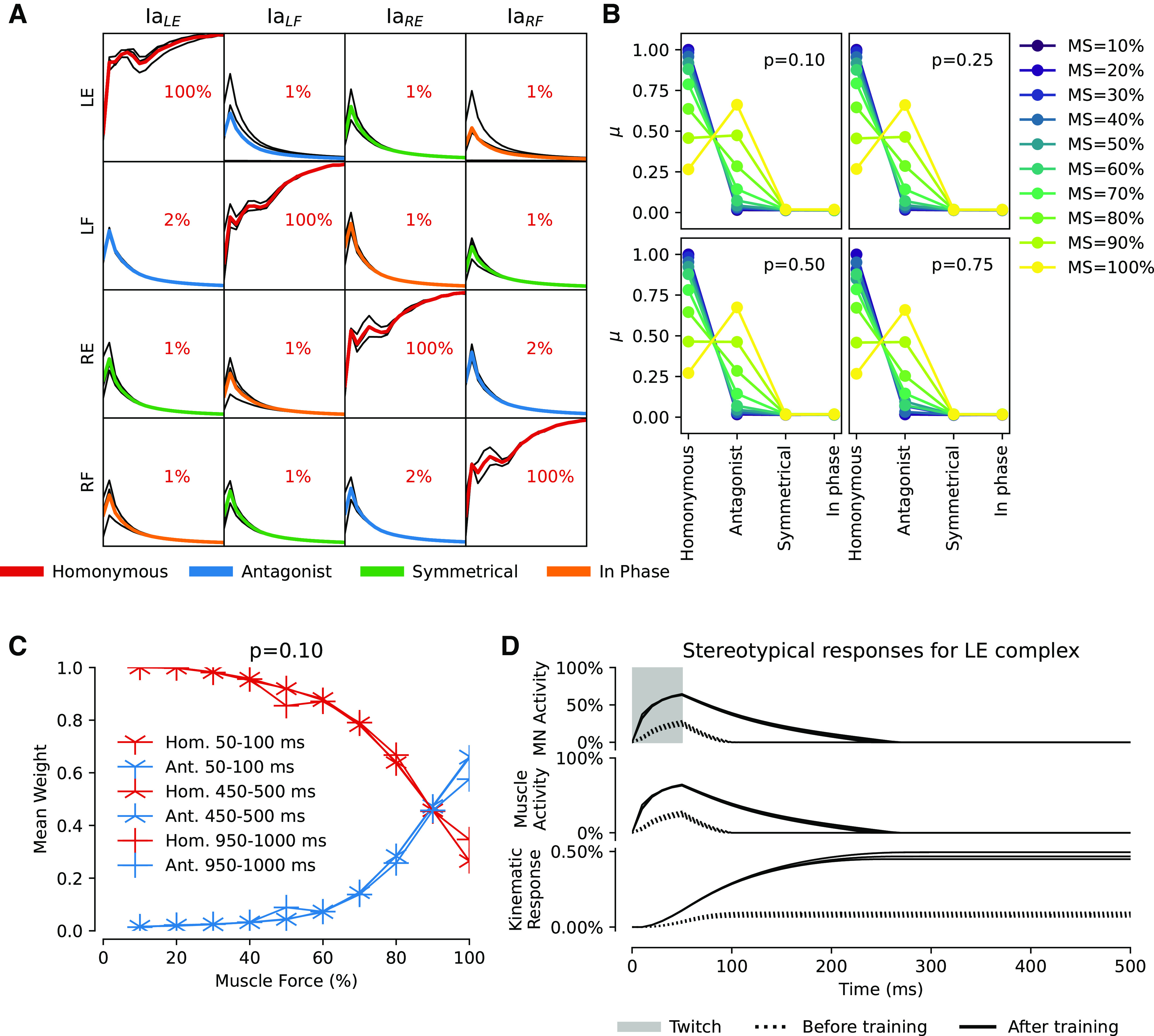
*A*: matrix of Ia-βMN synaptic weights during 20,000 s of training using the twitch APG with muscle strength at 10% for three different randomizations of initial weights. Each row illustrates one βMN, denoted by which muscle it innervates; incoming Ia synapses are organized in columns, where the subscript indicates the muscle of origin of the sensor. The colored mean lines indicate the functional relationship between the βMN and the sensor according to the legend below. *B*: summary of additional simulations with varying muscle strength (10%–100% with 10% increments as shown by legend to the right). Each 4-by-4 synaptic weight matrix has been summarized by functional relationship between βMN and sensor as identified in *A*. Each panel reports mean final synaptic weight (*n* = 3) as a data point for four different twitch probabilities p indicated. *C*: mean end weight for homonymous (red lines) and antagonists (blue lines) for different muscle forces (*x* axis) and twitch durations (different markers as indicated in legend); *P* = 0.1 for all simulations. Ia inversion is seen at 100% muscle force. *D*: evolution of motoneuron activity and corresponding kinematic response for the LE muscle in *A*. Top plot shows LE MN response to a 50-ms square pulse twitch, which is augmented by Ia feedback after training. Middle plot shows respective resulting LE muscle force. Bottom plot shows the kinematic response of the limb. The absolute amplitude of the response is small, but that is to be expected because the muscle force is set to 10% of max. Ia, primary afferent; LE, left extensor; LF, left flexor; MN, motoneuron; MS, muscle strength; RE, right extensor; RF, right flexor.

The synaptic weight learning of our first set of simulations with the twitch APG (*n* = 3, each made with different initial synaptic weights between the Ia sensors and the βMNs, 10% muscle strength and twitch probability of 0.1) quickly converged such that Ia-afferent feedback synapses for the homonymous muscle were potentiated whereas those from the nonhomonymous muscles were depressed (Fig. 6*A*, the grand mean synaptic weight of the diagonal was 1.0, SD = 7.5e-6), i.e., a similar synaptic weight matrix as observed for Ia-MN synapses in adult mammals (14). To evaluate the impact of such severely reduced muscle strength we ran additional simulations with incremented muscle strength, increasing by 10% up to the maximum of 100% (*n* = 3 for each setting). Not surprisingly the specificity of the homonymous Ia synaptic strength started to decline as the extrafusal muscles gained in strength. The mean homonymous Ia-MN synapse weight remained at a high weight until a level of around 60% muscle strength where a sharper decline started. At 90% muscle strength the antagonistic Ia-MN synapse weights were equal to the homonymous. A reversed connection scheme, where the antagonistic Ia-MN synaptic weight was higher than that of the homonymous, arose at 100% muscle strength. The increase in muscle force naturally produced larger limb movements (Fig. 4) that tended to unload the homonymous muscle spindle, reducing the feedback on which the Hebbian learning depends (Fig. 6*B*).

Both the frequency and duration of the twitches modulate the kinematic response. We re-ran the above simulations with the twitch probability set to 0.25, 0.50, and 0.75 (Fig. 6*B*). However, the results were nearly identical to the set of simulations with a twitch probability of 0.1, so twitch probability did not affect outcome. Next was the duration of the twitches, where a longer twitch would in theory give the muscle longer time to unload the muscle spindle. To explore this, we re-ran the above simulations once again, but with twitch durations of 450–500 ms and 950–1,000 ms. The twitch probability was in all simulations set to 0.1 (Fig. 6*C*). In summary the twitch duration did not alter the initial outcome seen in Fig. 6, *A* and *B*. This indicates that the precise nature of the muscle twitches did not play a crucial role in the outcome of the synaptic learning, whereas the relative strengths between intra- and extrafusal fibers did (Fig. 6*B*).

We also evaluated the behavior of the system before and after learning. In the untrained network, a 50 ms square pulse at 100% twitch amplitude was injected into the APG synapse of the left extensor motoneuron. The injection produced neuronal response as well as movement (Fig. 6*D*, dotted lines). After training, however, the same pulsatile injection produced a much more elongated neuronal response with a correspondingly elongated kinematic response of the limb (Fig. 6*D*, solid lines). Hence, training increased the capacity of the network to sustain movements long after the termination of the APG command. When tested with the phasic APG pattern before and after training, this effect resulted in stronger muscle impulses and more complex limb dynamics (Fig. 5), including the beginnings of oscillatory limb movements due to stretch reflexes (antagonistic response dynamics can be seen in Fig. 4*B*).

### Reduced APG Results in Less Distinct Connection Patterns

**Figure 7.**
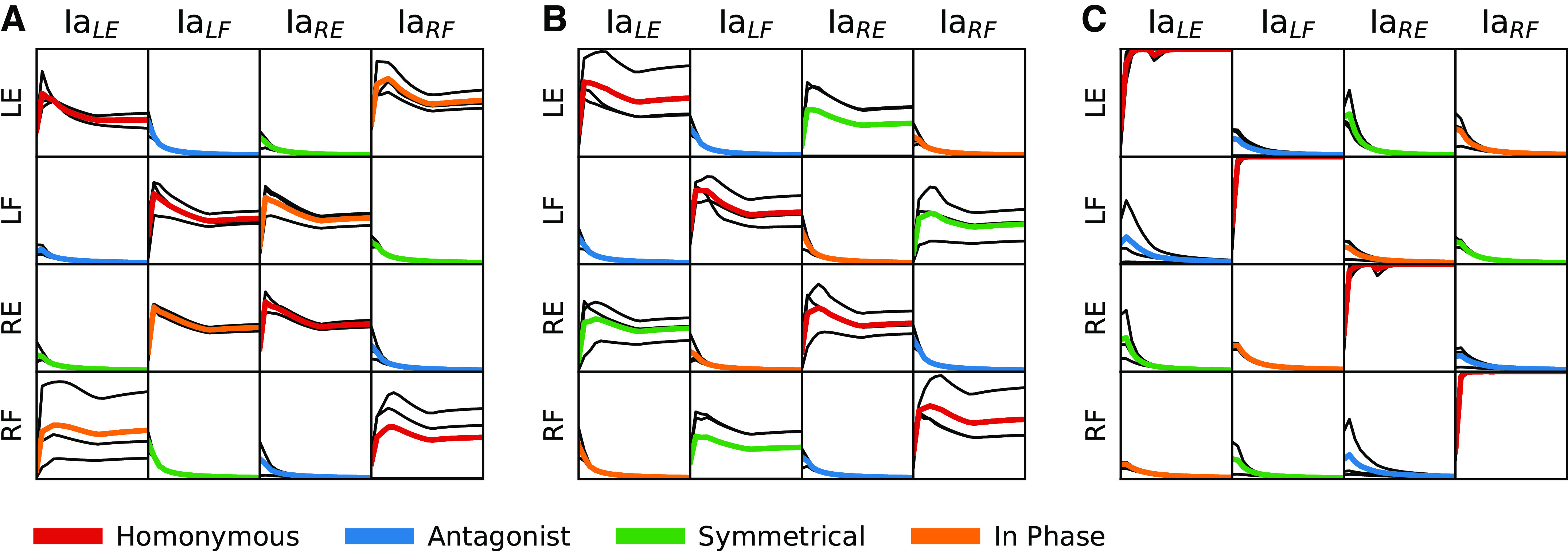
Synaptic weight matrices illustrating learning outcome induced after training with reduced twitch APGs. *A*: the twitch APG only generates in-phase movements. *B*: the twitch APG only generates symmetrical movements. *C*: the twitch APG never generates co-contraction. Each panel reports mean data from *n* = 3 simulations (colored line) and individual traces (black lines). Twitch probability for all simulations was 0.1; muscle strength was 10% of max. APG, activity pattern generator; LE, left extensor; LF, left flexor; Ia, primary afferent; RE, right extensor; RF, right flexor.

Although our twitch APG has little conceptual relation to a hypothetical CPG, CPG theory promotes the idea that there exists prewired neuronal circuitry that produces behaviorally relevant cyclic alternating activations of muscles that could support locomotion. Hence, by this definition a CPG would generate only a subset of the theoretically possible MN activation combinations. Therefore, we also tested reduced variants of the twitch APG where not all theoretically possible MN activation combinations occurred, with muscle strength set to 10% as above. Training with the twitch APG variant that produced only in-phase movements (see Fig. 2*C* and Fig. 7*A*) resulted in a moderate homonymous Ia-MN connectivity (*n* = 3, mean = 0.41, SD = ±0.044) plus an equal connectivity with the in-phase synergist in the contralateral limb (mean = 0.48, SD = ±0.045). Conversely, the twitch APG variant that produced only symmetrical movements (Fig. 2*C* and Fig. 7*B*) again resulted in a moderate homonymous Ia-MN connectivity (*n* = 3, mean = 0.53, SD = ±0.044) and was in this case also accompanied by a moderate Ia-MN connectivity (0.36, SD = ±0.05) between the extensors of each limb and between the flexors of each limb.

A less reduced variant of the twitch APG, in which only muscle co-contraction was forbidden, produced a synaptic weight pattern (Fig. 7*C*) that was similar to that of the nonreduced twitch APG (Fig. 6*A*). The homonymous projections (diagonals) were clearly potentiated (*n* = 3, mean = 1.0, SD = ±6.4e-14) and the remaining synaptic connections weakened to negligible.

### The Fusimotor Effect on the Ia Sensors Is Necessary for Learning

**Figure 8.**
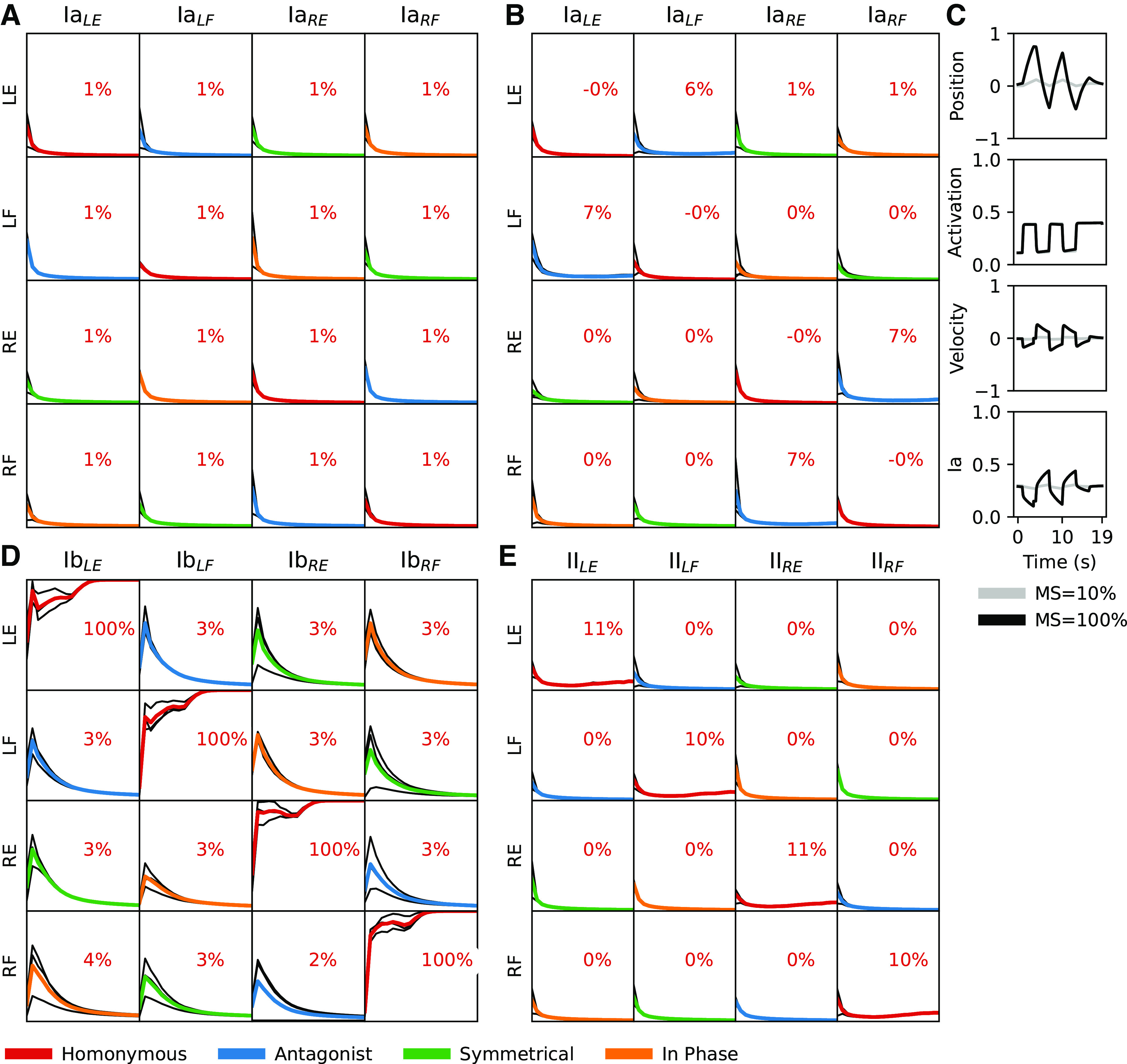
Learning outcomes after training using supplementary contexts. *A*: Ia sensor is left intact but the fusimotor of the motoneurons has been turned off, thereby simulating αMNs instead. Muscle strength was 10%; twitch duration 50–100 ms with a probability of 0.1. *B*: equal to context in *A* but with muscle strength set to 100% of max. *C*: example traces for limb position, muscle activation, muscle velocity, and Ia-activity for a set of predefined movements (equal to first column in Fig. 5 but using αMN as in *A* and *B*. *D*: Ia sensor feedback has been changed to Ib sensor feedback. *E*: Ia sensor feedback has been changed to II sensor feedback. LE, left extensor; LF, left flexor; Ia, primary afferent; MN, motoneuron; MS, muscle strength; RE, right extensor; RF, right flexor.

As a control, we eliminated all fusimotor effects of the MNs, effectively simulating an organism with only αMNs. With this adaptation of the Ia sensor, we ran a set of simulations (*n* = 3) with the muscle strength again set to 10% and twitch probability 0.1. In this case, the synapse-specific potentiation of the Ia-MN projections seen in previous simulations disappeared and all Ia-MN synapses trended toward zero strength (Fig. 8*A*). However, the only way to get a reasonable response from any Ia sensor in this case is through overt limb motion. This requires more muscle force, so we ran an additional set of simulations (*n* = 3) with the muscle strength set to 100% (Fig. 8*B*). In this case the homonymous synaptic weights were depressed rather than potentiated and there was some potentiation of the Ia synapses onto the antagonist MN. We confirmed that the plant kinematics during APG input was largely preserved (Fig. 8*C*). In fact, all Ia sensors had substantial activity during the training but these activations were not well correlated with the activation of the muscles.

### Nonbiological Sensor Input and Hebbian Learning

For curiosity we also tried exchanging the Ia sensors with the Ib and II sensors, even though neither of these proprioceptors is known to make monosynaptic connections to motoneurons. We ran *n* = 3 simulations for each variant while keeping the remaining parameters the same (muscle strength at 10%, twitch duration 50–100 ms, twitch probability at 0.1). The simulations using Ib quickly converged to a strong homonymous synaptic strength, while decorrelating the remaining inputs (Fig. 8*D*). This is not surprising because any tension in the extrafusal fibers will excite the Golgi tendon organs and thus generate sensory feedback that can drive Hebbian learning. At higher muscle forces, Ib activity tracks homonymous MN activity even more, whereas fusimotor-driven Ia activity starts to be opposed by the resultant extrafusal muscle shortening (Fig. 6, *B* and *C*).

The simulations using group II as the sensor input produced much weaker but still selective homonymous connectivity. The fusimotor component of βMN activity has a weaker effect on the II than the Ia ending (see *Eq. 6*) and the II activity is more dependent on muscle length, which tends to shorten more when the active muscle is stronger.

## DISCUSSION

### Nature versus Nurture

Selective formation of Ia-MN connectivity has been attributed to *1*) genetically specified cell-cell recognition molecules [particularly for homonymous projections (9, 60)], *2*) positional cues in ventral horn [particularly for refinement of heteronymous circuits (61, 62)], and *3*) activity-dependent self-organization as suggested here. The plausibility of the latter was rejected ∼30 yr ago largely because curare block of muscle activity in fetal chick (13) and disorganization of muscle activity following ventral root transection in bullfrog (63) both resulted in relatively normal patterns of Ia-MN projection. The original interpretation of those experiments, however, must be reexamined in light of more recent understanding. The prevalence of βMNs in amphibia (64, 65) and birds (66) and their persistence in mammals (22) was under-appreciated then and was not mentioned. The importance of gap-junctions in generating spontaneous fetal activity in and among motor pools and their organization according to anatomical proximity of MN somata were unknown (23). Complete curare block of extrafusal muscle fibers does not completely abolish intrafusal muscle activation (particularly of the chain fibers that can directly drive Ia afferents) but that was discovered later (67). In the fetal chick preparation, spontaneous, synchronous activation of MN pools including βMNs would provide strong Hebbian reinforcement of homonymous Ia-MN projections even when the extrafusal muscle was paralyzed. The observation that the homonymous EPSPs were actually larger than normal could be explained by the absence of extrafusal unloading, as observed in our model system. The weaker heteronymous projections that formed in both normal and curarized chicks were selective for MN pools that overlap in the ventral horn (68, 69), hence likely to share at least weak gap junctions. The resected ventral roots of the bullfrog were known to reinnervate muscles in random patterns that disrupted coherent limb motion but a βMN that found its way into one muscle would likely innervate both extrafusal and intrafusal muscle fibers in the same muscle, restoring the muscle-specific Hebbian cue. As demonstrated here, the βMN mechanism would enable classical Hebbian learning, whereas overt limb motion requires an unphysiological reverse Hebbian mechanism to generate the adult Ia-MN connectivity (17).

The present study provides a basis for functional self-organization of the Ia-MN connectivity in the spinal cord based on sensorimotor experience resulting from random activations of these MNs during early development (i.e., nurture). The selective projection of Ia afferents onto the MNs of their homonymous parent muscle did not require any a priori knowledge of which MNs innervated which muscles. Instead, it emerged from the random activation of the musculoskeletal anatomy, but only when that included co-innervation of both intrafusal and extrafusal muscle fibers, as provided by βMNs (as opposed to independent α and γMNs). These results are in line with studies into myotonic specificity where recovery of functional behavior occurred after cross connection of peripheral nerve fibers (70). Indeed, βMNs are known to be widely present in mammals (71), whereas mature γMNs are present only in adult mammals and only gradually develop from perinatal stages and onwards (21, 72). Hence, the effects observed here are likely to be dependent on βMNs. However, similar effects “could” be achieved if α and γMN activations were tightly coupled by gap junctions at this early stage of fetal development. It is worth noting that α and βMNs are likely to be so-coupled, which would result in homonymous spindle afferent synapses onto both α and βMNs in a given motor nucleus.

The developmental role for βMNs proposed here begs the question of how the somewhat weaker but still common heteronymous Ia-MN synapses form between synergistic muscles. As noted earlier, at least some of that may be attributable to anatomical proximity of those motor pools in the ventral horn and/or weak gap junctions between synergist MNs. Some of the known patterns in adult mammals, however, appear to be more closely related to functional synergy in the limb rather than proximity in the ventral horn (14). The Oropod is not suited to explore this due the absence of synergistic groups of muscles. Nevertheless, the results presented here suggest that a strong muscle twitch into a muscle would unload both the homonymous and synergist spindles; without the driving βMN input the synergists would fail to potentiate the appropriate synapses. However, along the same line of reasoning, a similar twitch into an antagonist muscle would introduce a positive muscle stretch in all synergists, thereby activating their Ia afferents. If their homonymous synapses resulted in reflexive activation of their MNs (as demonstrated in Fig. 4*B*), the overlapping afferent and efferent activity among the synergists could potentiate the heteronymous Ia-MN synapses. Note that this could happen only at a developmental stage when the growing strength of the extrafusal muscle fibers starts to produce overt limb motion (see Fig. 6*C*). Such motion would enable the development of the fairly widespread interconnectivity among partial synergists (14, 15) to reflect their functional, as opposed to anatomical, relationships in the limb.

Our results indicate a dependence upon the interplay between the relative strength of extrafusal and intrafusal muscle fibers, which is likely to shift during development. For example, at birth, or hatching, terrestrial animals must suddenly deal with gravity. Given that skeletal muscles become stronger with exercise, we made the assumption that in early development intrafusal fibers are ‘stronger’ than extrafusal fibers (30). This implies that whenever there is βMN activation, the Ia sensors will be activated despite the unloading effects of extrafusal force generation. This is a necessary condition for the learning we describe here to work, which will require future experimental testing. Another prediction from our model is that this phenomenon is probably dramatically reduced over time after birth, during which time extrafusal fiber strength has indeed been observed to increase tenfold or more (30). At that point in development, however, the Ia-MN synapse may be less plastic and/or the γMN system may start to provide the adult patterns of alpha-gamma coactivation that tend to accompany behaviors in which the muscles are expected to shorten (18).

The absolute rule that Ia but not Ib afferents connect monosynaptically to MNs is often interpreted as an indication of a type-specific affinity, but it may be explained by a critical period in the development of “any” monosynaptic afferent input to MNs instead of a genetic difference in the afferents themselves. When substituting the Ia stretch sensor with the Ib force sensor in our model system, the resulting connectivity matrix was as specific (Fig. 8*D*) as for the Ia (Fig. 6*A*). Nevertheless, monosynaptic Ib connections to MNs are not found in adult animals. Notably, muscle spindles with equatorial Ia afferent terminals are found as early as *E19–20* in the rat (73), while the entanglement of Ib nerve endings in the collagen bundles of Golgi tendon organs (GTO) is not observed until the first postnatal week (74) and they continue to grow until *week 3*. By that time, our model indicates that continued plasticity in the Ia-MN synapse becomes counter-productive and should be prevented. A further suggestion of lack of type-specificity between Ia and Ib afferents comes from their tendency to reinnervate the “wrong” receptors during regeneration following peripheral nerve repair in adults (75), which would result in monosynaptic projections between functionally Ib afferents and homonymous MNs.

### Neuronal Model

The neuronal model used in this article (25) provides a simplified neuronal calcium dynamics model with a continuous output signal rather than spikes. Oja’s learning rule (52) that we adapted for our model generates synaptic potentiation or depression according to the relative timing of calcium transients in the pre- and postsynaptic compartments of the synapse. We assumed that the postsynaptic calcium concentrations reflected simply the level of activity of the postsynaptic cell, but this is not always the case. For example, the inherent calcium dynamics of cuneate nucleus neurons causes the postsynaptic calcium to reflect a time derivative of the underlying membrane potential (26). If such a situation obtained in MNs, the learned synaptic modifications would depend on more dynamic aspects of MN activation; in the limit this would resemble a spiking neural model. Unfortunately, little seems to be known about dynamic calcium gradients in early embryonic spinal MNs, so we elected to use the simplest assumption.

Both the synaptic weight matrices resulting from the learning process and the end synaptic weights of the diagonal in these matrices were remarkably stable over a large range of initial weights (*n* = 3; see Fig. 6*A*). This stability was also evident over a large range of muscle strengths, twitch probabilities and twitch durations (Fig. 6), and also when considering nonbiological sensor input to the motoneurons (Fig. 8, *D* and *E*). Considering the robustness of our neuronal learning model, the end results may be robust across a further range of implementations.

The low dimensional biomechanics and network we studied here can be seen as quite trivial in relation to the biological counterparts. Here we focused on illustrating the principle that self-organizing spinal cord circuitry could be made to work. Future studies are needed to show if the system could work similarly with a higher number of degrees of freedom in the plant, with partial synergists such as multiarticular muscles and/or with a larger network and with other sensory modalities and the interneurons that convey them to MNs.

### Comparative Development

If the synaptic plasticity of the Ia-MN projections remained unchanged during development, the specificity of the homonymous loops appears likely to decline as the muscles strengthen and the random twitch APG transitions to the more phasic APG patterns that we tested as representative of CPG drive (effectively more extreme versions of the restricted twitch APGs presented in Fig. 7). Such an outcome could be avoided by introducing a limited critical period in this synaptic plasticity and/or developing γMN activity whose intrafusal effects counter these kinematic effects. The timing of each of these hypothetical changes during fetal and perinatal development is likely to be critical for successful ontogeny, so might be expected to be embedded in genetically specified general rules for development. Failure to follow any single step in this choreographed ontogeny would likely give rise to a wide range of secondary changes in the circuitry that would be observed as pathological phenotypes in the adult.

If fetal and perinatal development depends on timed sequences of events, how much recovery will be possible in the event of injury to either musculoskeletal or neural components at various times of life? Extraordinary adaptation to supernumerary muscles and limbs has been observed in amphibia but appears to depend on age. Such adaptation occurs quickly in larvae but slower in older animals (76). Even among species with similarly freely swimming larval stages, there are substantial differences in the consequences of muscle paralysis on subsequent behavioral deficits (77).

A counterpoint can be found in the fact that an embryo confined in ovo has a quite restricted space in which to move (78), resulting in a restricted posture of the embryo during development (79). Such restrictions might have some influence on the outcome of development (79), but the end result is still very much functional (80). Such restrictions do not negate that the outcome requires extensive learning, rather that the sensitivity to posture and movement may be somewhat different in these species. Thus, even though our twitch APG in theory exposes our system to every combination of activations in every posture, which might be a richer experience than for many biological counterparts, the increased richness due to space and overt movement does not seem to be the main source of our results. Rather we see correct homonymous connectivity arising due to relatively stronger intrafusal versus extrafusal effects on spindles regardless of twitch duration (increased movement, Fig. 4).

In adult mammals, nerve crossings and muscle transpositions result in persistence of the original neural circuitry and function (81–83), whereas muscle transpositions in neonatal cats result in changes in at least some muscle function and cutaneous reflexes (84). Congenital duplication of the forearm and hand in man has been described with relatively intact sensorimotor function (85). Absence of sensory feedback during development in humans results in dysfunctional muscle use (86). All of these suggest that there are critical periods of development during which the spinal circuitry is sensitive to patterns of sensory input.

What we have demonstrated here is that muscle-specific Ia connectivity “can” develop with appropriately configured type-specific rules, namely the presence of βMNs linked by tight junctions and prone to spontaneous twitches and a critical period of synaptic plasticity while the extrafusal muscles gain strength. In other work underway, we are exploring whether this general principle can be extended to the interneuronal circuits of the spinal cord.

Reliance on nurture rather than nature during ontogeny, wherein the physical properties of the biomechanical plant can be imprinted on the NN without any a priori knowledge, is potentially important to understand the successful evolution of new species. Such evolution is driven largely by mutations that initially affect musculoskeletal mechanics. An individual with a potentially useful musculoskeletal mutation must first adapt to that mutation and survive to reproduce before any subsequent coevolution of the neural circuitry can occur. If ontogenetic connectivity depended on the phylogenetic a priori “knowledge” embedded in chemically hardwired connectivity loops between muscles, that knowledge would be obsolete and potentially fatal with every new musculoskeletal mutation. In direct contrast, an evolutionary lineage that utilized a general solution to the control problem of novel biomechanics would gain a significant advantage for the evolution of new species.

## GRANTS

This work was supported by the European Union Grant FET 829186 ph-coding (Predictive Haptic COding Devices In Next Generation interfaces), and the Swedish Research Council (Project Grant No. K2014-63X-14780-12-3).

## DISCLOSURES

No conflicts of interest, financial or otherwise, are declared by the authors.

## AUTHOR CONTRIBUTIONS

J.M.D.E., H.J., and G.E.L. conceived and designed research; J.M.D.E., A.M.J., M.K., and J.H. performed experiments; J.M.D.E., H.J., and G.E.L. analyzed data; J.M.D.E., H.J., and G.E.L. interpreted results of experiments; J.M.D.E., H.J., and G.E.L. prepared figures; J.M.D.E., H.J., and G.E.L. drafted manuscript; J.M.D.E., H.J., and G.E.L. edited and revised manuscript; J.M.D.E., A.M.J., M.K., J.H., H.J., and G.E.L. approved final version of manuscript.
